# Assessment of differential diagnostic skills of physiotherapists related to the cervical spine - approaches to improving effectiveness: observational, cross-sectional study

**DOI:** 10.1186/s12909-025-07682-x

**Published:** 2025-07-16

**Authors:** Kinga Nákity, Blanka Bernadett Kasza, Barbara Bianka Tatár, Mónika Szűcs, Dávid Kis, Pál Barzó, Andrea Domján

**Affiliations:** 1 Albert Szent-Györgyi Medical School, Doctoral School of Inderdisciplinary Medicine, University of Szeged, Semmelweis street 6, Szeged, 6723 Hungary; 2 Albert Szent-Györgyi Health Centre, Department of Physiotherapy, University of Szeged, Szeged, Hungary; 3 Faculty of Health Sciences and Social Studies, Department of Physiotherapy, University of Szeged, Szeged, Hungary; 4 Albert Szent-Györgyi Medical School, Faculty of Science and Informatics, University of Szeged, Szeged, Hungary; 5 Albert Szent-Györgyi Health Centre, Department of Neurosurgery, University of Szeged, Szeged, Hungary

**Keywords:** Decision-making, Differential diagnostic knowledge, Cervical spine, Critical medical cases, Musculoskeletal, Warning signs, Red flags, Physiotherapy

## Abstract

**Background:**

Cervical disorders are a significant cause of disability worldwide, often presenting with pain and symptoms affecting the musculoskeletal system and other organs. It is essential for healthcare professionals to accurately identify and comprehend the underlying causes of these potentially life-threatening conditions. This study aimed to assess the diagnostic knowledge and clinical decision-making abilities of physiotherapists in Hungary regarding serious health conditions in patients with cervical disorders and the factors influencing these decisions.

**Methods:**

Data were collected through an electronic questionnaire containing demographic information, educational background, and professional experience. The second section includes excerpts from eight case reports on diagnosing cervical complaints from international literature. A cohort of 128 physiotherapists (114 female and 14 male; mean age: 34.65 ± 8.88 years; 101 BSc degrees and 27 MSc/PhD degrees, average of 9.73 ± 7.82 years of professional practice) who completed their higher education in Hungary were recruited for participation. Statistical analyses were performed using R software version 4.0.2. The primary outcome was correct decision-making, measuring its association with years of practice, clinical experience, educational level (BSc, MSc/PhD), and postgraduate training in cervical spine management.

**Results:**

61.7% of therapists identified the appropriate treatment for musculoskeletal cases, while only 22.7% recognized critical signs and symptoms. Participants with postgraduate training in neck conditions (odds ratio 1.25–7.99, *p* = 0.014) and those treating orthopedic (odds ratio 0.18–0.99, *p* = 0.047) and trauma cases (odds ratio 0.11–0.65, *p* = 0.004) were significantly more effective at recognizing critical cases.

**Conclusion:**

Our findings emphasize the need for ongoing education and training to recognize cervical spine red flags. These results align with global literature and highlight the importance of improving diagnostic knowledge in undergraduate programs. These results underscore the significance of extensive professional experience and advanced education within the discipline.

**Clinical trial number:**

Not applicable.

**Supplementary Information:**

The online version contains supplementary material available at 10.1186/s12909-025-07682-x.

## Introduction

Spinal disorders impact a substantial portion of the global working-age population, imposing significant burdens on individuals, families, and societies [1]. Cervical spine disorders are the fourth most prevalent cause of disability worldwide [2]. The regular use of healthcare services by individuals with cervical or lumbar spine conditions underscores the importance of evidence-based treatment, which should be grounded in three fundamental dimensions: safety, comfort, and efficiency [3–6].

Patients with spinal disorders sometimes present with severe pathological symptoms [7]. An in-depth history and physical examination provide clinicians with essential information for devising an effective treatment plan. Identifying red flags is crucial for practitioners [8]. Red flags indicate the need for immediate patient evaluation, but conservative management should be implemented in the absence of red flags [9–11]. An international framework of red flags for identifying potentially serious spinal pathologies [12] is available for physiotherapeutic spinal assessment and management; the knowledge and application of this framework are imperative for healthcare professionals.

The structure and function of the cervical spine differ from those of other spinal segments. The cervical region permits more movement than other spinal regions but is structurally more complex, has less bony stability, and is therefore more susceptible to injury [13]. Therefore, physicians and physical therapists must approach the examination and treatment of the cervical spine differently from those of the lumbar segment [7]. Many cervical spine issues arise from musculoskeletal (MSK) pathologies, including myofascial pain syndrome, radiculopathy from disk degeneration, spinal stenosis, myelopathy, various listhesis-related problems, and traumatic injuries [14]. Non-MSK conditions, including tumors, infections, and diseases of the cardiovascular, respiratory, gastrointestinal, endocrine, and rheumatologic systems [15, 16], may also induce neck pain [17]. Guidelines for identifying the underlying pathologies of neck pain are summarized in the review by Feller et al., 2024 [18]. Patients with MSK complaints may seek consultation from general practitioners and various healthcare professionals and providers. Individuals with persistent acute or chronic MSK issues may initially present to physiotherapists. According to a 2012 survey conducted by Bury and Stokes, access to physical therapists is typically facilitated through indirect means in 15 countries within the World Confederation of Physical Therapists (WCPT) after medical examination and referral. In Hungary, according to the Ministry of Human Resources Decree 18/2016 (VIII.5.) [19], physiotherapists determine the appropriate interventions and independently manage and assume responsibility for their professional activities based on the medical diagnosis and their assessment of the patient. Hungarian legislation does not, in principle, permit direct access to physiotherapy, unlike in several European and overseas countries, such as Norway, Finland, Sweden, the Netherlands, Belgium, the United Kingdom, Ireland, Portugal, and Estonia, as well as several states of the USA, Canada, and Australia [20, 21].

In Hungary, physiotherapists in outpatient government-funded care treat patients based on a doctor’s referral. However, in private practice, a referral is not required for physiotherapy, and private services have become increasingly common and widely used. Autonomous physiotherapists require proficiency in identifying clinical signs and symptoms that pose life-threatening risks to patients [10, 22]. According to the WCPT guidelines, when a physiotherapist identifies findings outside their expertise during physiotherapeutic assessment and diagnostic evaluation, the physiotherapist should inform the patient and refer the patient to an appropriate specialist or physician [23, 24]. Sound clinical decision-making relies on the cognitive competence of differential diagnostic reasoning and the understanding and application of these processes [11, 25].

Several recent studies have evaluated the diagnostic and clinical decision-making skills of physiotherapists in managing MSK disorders. Research has shown that physiotherapists are well-equipped to assess, diagnose, and manage MSK conditions with a level of accuracy comparable to that of physicians. Cattrysse et al. (2024) conducted a scoping review of 22 studies (5 systematic reviews + 17 primary studies) assessing the quality of direct-access physiotherapy for MSK disorders from the perspectives of the patient, the provider, and society [26]. This review highlighted the effectiveness and safety of physiotherapist-led MSK management under a direct-access model. A systematic review found that advanced-scope physiotherapists demonstrate high diagnostic accuracy, appropriate triage, and effective management of MSK disorders [27]. Moreover, multiple studies have explored the differential diagnostic decision-making capabilities of graduate physiotherapy students and experienced practicing physiotherapists, focusing on their ability to recognize warning signs and symptoms (red flags) [28–32]. However, the differential diagnostic decision-making abilities of physiotherapists who have graduated domestically and are currently practicing within the Hungarian healthcare system have not been studied.

The primary objective of this study was to evaluate the proficiency of physiotherapists practicing in Hungary, including competency and education in recognizing the warning signs and symptoms associated with MSK disorders of the cervical spine and critical medical (CM) conditions, and assess the suitability of therapeutic interventions in MSK cases. In addition, we aimed to identify variables that influence the differential diagnostic reasoning and decision-making processes of the participating therapists.

## Methods

### Participants and setting

**Fig. 1:**
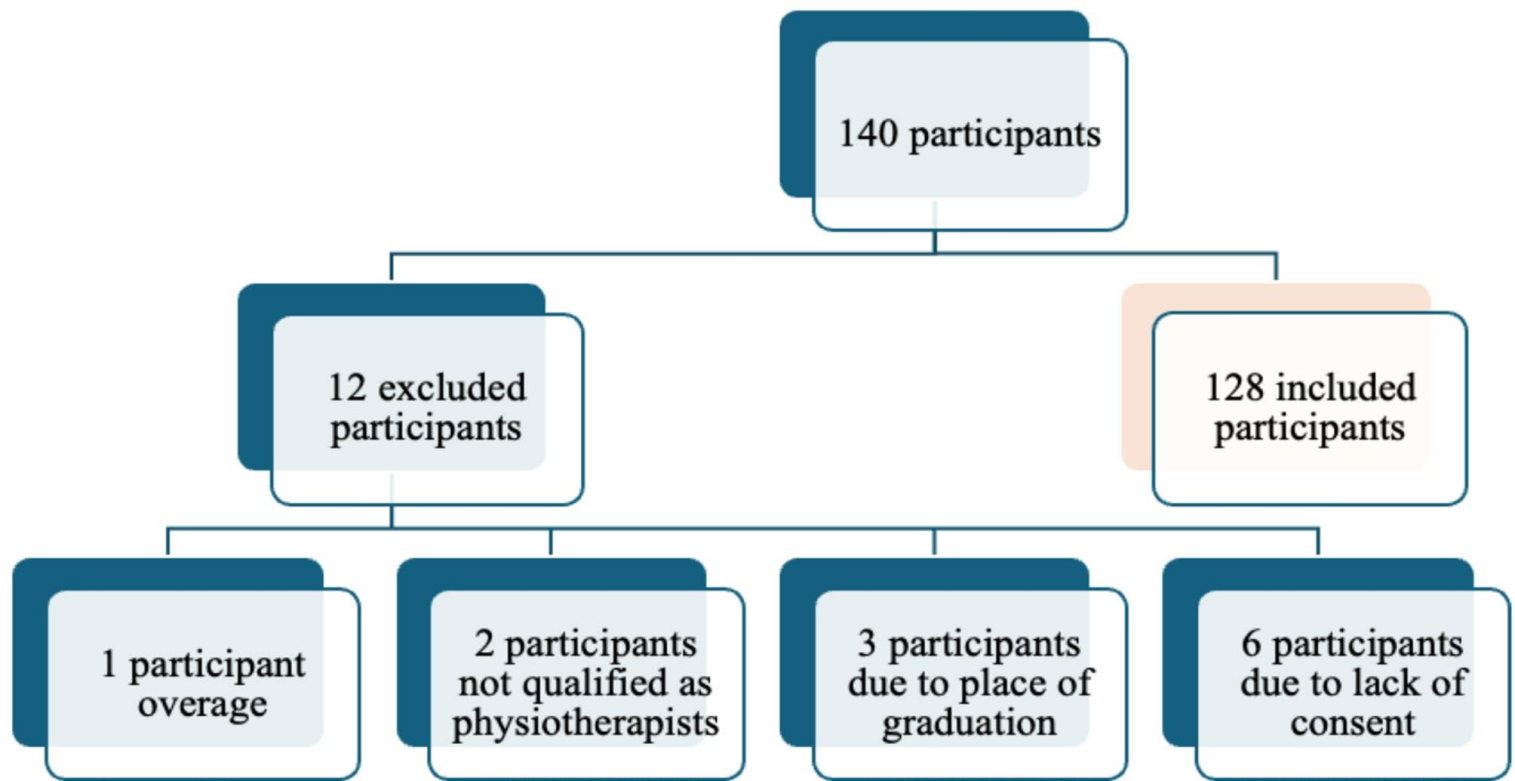
Flow chart of excluded participants

Participants with physiotherapy qualifications were recruited for the study via online platforms with the approval and support of the Chamber of Hungarian Health Care Professionals (MESZK - www.meszk.hu) and the Association of Hungarian Physiotherapists (MGYFT -www.mgyft.hu). The eligibility criteria were as follows: aged 25–65 years, completed higher education in Hungary, earned at least a bachelor’s degree in physiotherapy, and were currently employed in Hungary. Individuals who were no longer actively practicing or who failed to meet the inclusion criteria were excluded from the study (Fig. 1).

Survey data were gathered electronically with a questionnaire via EvaSys software. Therapists completed the questionnaire once they accessed the website www.mgyft.hu from June 23 to September 10, 2022. The study was approved by the Hungarian Medical Research Council (ethics approval number IV/3426-1/2022/EKU). All the participants provided informed consent to participate in the study.

### Survey development

The following participant data were collected: demographics, education, work experience, and workplace (Appendix 1). The questionnaire presenting the case studies and treatment options was developed on the basis of the research methodologies of Jette et al., 2006 and Ladeira, 2018. The case studies (designated CS 1–8) included condensed case histories and physical examination results from eight cervical spine cases, including two cases with MSK complaints [33–35] in the background (CS 4, CS 6) and six cases with life-threatening signs and symptoms [36–42] (CS 1, CS 2, CS 3, CS 5, CS 7, CS 8) (Appendix 2). After reviewing the case studies, the respondents were asked to select 1–5 intervention options from 18 treatment options listed for managing the case. Intervention options included various physiotherapeutic treatment methods, which are based on international professional protocols [43, 44]. Participant responses were categorized into two recommendation groups: (1) administer a physiotherapeutic intervention or (2) provide advice or refer to another healthcare professional/physician (Appendix 3). Suggestions and referrals were deemed correct for cervical medical (CM) cases, and interventions were considered appropriate for musculoskeletal (MSK) cases.

### Content validity of the case studies

The selected case studies were case reports published in English in international journals. These cases focused on cervical spine complaints because recognizing the pathomechanism of cervical spine complaints is crucial for health professionals. The cases were retrieved from the PubMed, Science Direct, Medline, and Scopus databases via the following search terms: “differential diagnosis,” “differential diagnostic knowledge,” “neck pain,” “cervical spine,” “referral pain,” “fracture,” “myelopathy,” “cardiovascular,” “cervical radiculopathy,” “arterial vertebral dissection,” “myofascial pain syndrome,” “atlantoaxial instability,” and “metastatic tumor.” Each CM case included warning signs, symptoms, and clinical data gathered from general practitioners, specialists, and physiotherapists, as deemed appropriate. The case reports were translated into Hungarian by two physiotherapists with Master’s degrees in physiotherapy. The cases were subsequently reviewed by a physiotherapist with a PhD in physiotherapy and a postgraduate specialist with a degree in medical translation and interpretation. The final review was conducted by a multidisciplinary team of physicians (neurologists, neurosurgeons) and physiotherapists.

### Statistical analyses

Statistical analyses were performed via R software (version 4.0.2). Descriptive statistics were used to characterize the subjects and treatment decisions and their distribution relative to the case reports. Treatment decisions were defined as “provide advice, refer to another healthcare” or “physiotherapeutic intervention” (Appendix 3). A referral in addition to treatment was classified as an intervention. The results are presented as the means ± SDs and frequencies (%). Factors influencing decisions in the MSK and CM categories and their relationships were analyzed via logistic regression. The odds ratios (ORs) and corresponding confidence intervals (95% CIs) were calculated, *p* ≤ 0.05 was considered significant. Associations between correct decision-making and the number (No.) of years of practice, clinical experience, educational level (BSc, MSc/PhD), and postgraduate training (PGTr) in cervical spine management were determined. These associations were examined for the MSK and CM categories and separately for each case. For cases in the CM category, decisions were considered acceptable if the participant gave the correct answer for at least three of the six cases. For the MSK category, a response was acceptable (Acc) only if the respondent correctly rated both cases. All other decisions were unacceptable (UAcc) in both categories. The STROBE (Strengthening the Reporting of Observational Studies in Epidemiology) guidelines were used to ensure the reporting of this observational study [45].

## Results

### Participants and descriptive data

The questionnaire was completed by 140 volunteers, and 128 responses (34.65 ± 8.88 years; 114 females and 14 males) were processed after applying the exclusion criteria. As shown in Tables 1 and 78.91% of the physiotherapists (101 persons, age: 35.22 years ± 9.30 years, work experience 10,04 ± 8,17 years) held a bachelor’s degree (BSc), and 21.09% (27 persons, age: 32.46 ± 6.1, work experience: 8.51 ± 6.61 years) held a master’s degree (MSc) or a PhD (26 persons MSc, 1 person PhD). All participants graduated between 1987 and 2022. The participants had a minimum of 1 and a maximum of 34 years of work experience, and 60.9% of the participants completed postgraduate training in cervical spine treatment, including the McKenzie method (45.57%), manual therapy methods (34.18%), and cervical spine stabilization training (31.65%). Fewer than 10% of the participants completed soft tissue mobilization techniques and postgraduate training for treating cervico-craniomandibular dysfunction. Approximately two-thirds (68.8%) of the therapists treated patients with indirect access, i.e., via specialist referrals.

Cervical complaints represented less than 30% of daily patient care for most (90.62%) participants; only 9.38% of the therapists treated patients with cervical symptoms for more than 30% of their work time. Two-thirds of the respondents (67.2%) had experience in orthopedics, 61.7% had experience in traumatology, and less than 50% had experience in neurology and neurosurgery, pediatrics, surgery, rheumatology, and other clinical areas.

**Table 1:** Characteristics of participating physiotherapists (*N* = 128)

	Frequency (%)	Mean (SD)	
**age**			34.648 (8.882)
**sex**			
female male	114 (89.1%) 14 (10.9%)		
**Educational level**			
BSc	101 (78.9%)		
* age* * work experience*			35.22 (9.30) 10.04 (8.17)
MSc/PhD	27 (21.1%)		
* age* * work experience*			32.46 (6.1) 8.61 (6.61)
**Work experience**			9.727 (7.822)
**Postgraduate training**			
yes	78 (60.9%)		
no	50 (39.1%)		
**Access**			
yes	88 (68.8%)		
no	40 (31.2%)		
**No. of patients with neck problems**			
10%	52 (40.6%)		
10-30%	64 (50.0%)		
30%<	12 (9.4%)		
**Clinical experience**			
orthopedics traumatology neurology neurosurgery pediatrics surgery rheumatology other	86 (67.2%) 79 (61.7%) 57 (44.53%) 29 (22.66%) 12 (9.38%) 23 (17.97%) 16 (12.5%) 21 (16.41%)		

### Responses to the MSK and CM cases

Seventy-nine (61.7%) respondents made appropriate treatment decisions in both MSK cases (CS 4, CS 6), and 22.7% of the respondents (*n* = 29) made appropriate treatment decisions in the CM cases (CS 1, CS 2, CS 3, CS 5, CS 7, and CS 8). No significant differences in treatment decisions were detected for MSK cases according to the participants’ education levels. For MSK cases, 59.4% of physiotherapists with a BSc degree made appropriate treatment decisions, and 70.4% of physiotherapists with an MSc degree made appropriate treatment decisions.

The percentage of participants who recognized the signs and symptoms of a serious health problem in the CM cases was low, regardless of the respondents’ level of academic education. (Table 2). Most (67.9%) participants with postgraduate training in cervical spine management chose appropriate treatment methods for cases in the MSK category, slightly exceeding the proportion of correct decisions in the overall sample (61.7%). Similarly, the correct decision rate for the CM category was 26.7%, which was slightly higher than the overall rate (22.7%).

On the basis of all MSK and CM case responses, decision-making was not significantly influenced by education level, access mode, or continuing education on the cervical spine. However, the evaluation of decision-making for each case revealed that postgraduate training affected decision-making specific to the neck (95% CI 1.25–7.99, *p* = 0.014). In addition, all CM decisions were positively influenced by therapists working with orthopedic (95% CI 0.18–0.99, *p* = 0.047) and traumatic (95% CI 0.11–0.65, *p* = 0.004) patients.

**Table 2:** Appropriate treatment decision in MSK and CM cases

	Frequency (%)	OR (95% CI)	*p*-value	
**MSK cases**				
**Degree**				
BSc	60 (59.4%)	-		
MSc	19 (70.4%)	1.62 (0.67–4.26)	0.300	
**Access**				
non-direct	29 (72.5%)	-		
direct	50 (56.8%)	0.50 (0.21–1.10)	0.094	
**Postgraduate training**				
no	26 (52.0%)	-		
yes	53 (67.9%)	1.96 (0.94–4.09)	0.072	
**CM cases**				
**Degree**				
BSc	24 (23.8%)	-		
MSc	5 (18.5%)	0.73 (0.23–2.01)	0.564	
**Access**				
non-direct	8 (20.0%)	-		
direct	21 (23.9%)	1.25 (0.52–3.29)	0.629	
**Postgraduate training**				
no	8 (16.0%)	-		
yes	21 (26.9%)	1.93 (0.81–5.04)	0.154	

### Factors influencing the decision-making process

The years of work/professional experience significantly influenced the recognition of the two CM cases and decisions about the required intervention. The warning signs and symptoms of dens fracture (CS 1) and cervical myelopathy (CS 2) were recognized by a high proportion of therapists (Table 3).

**Table 3:** Impact of years of work experience on decision-making

	OR (95% CI)	*p*-value
CS 1	1.07 (1.02–1.14)	0.015*
CS 2	1.09 (1.03–1.16)	0.006*
CS 3	0.97 (0.83–1.09)	0.699
CS 4	1.03 (0.98–1.09)	0.282
CS 5	1.03 (0.99–1.08)	0.143
CS 6	0.99 (0.93–1.05)	0.674
CS 7	1.05 (1.00-1.10)	0.067
CS 8	1.04 (1.00-1.10)	0.076

*p-value less than 0.05

The number of years of practice significantly influenced the selection of the appropriate treatment in these cases. Participants with clinical experience in orthopedics and traumatology also made appropriate treatment decisions about the cervical myelopathy case (CS 2), the atlantoaxial instability case (CS 7) and the metastatic tumor case (CS 8). Experience in trauma significantly affected decisions about dens fracture case (CS 1) (Table 4).

**Table 4:** Effect of clinical experience on the proportion of correct decisions in different cases

	Orthopedics		Traumatology	
	OR (95% CI)	*p*-value	OR (95% CI)	*p*-value
CS 1	0.56 (0.24–1.21)	0.147	0.45 (0.20–0.97)	0.046*
CS 2	0.23 (0.07–0.71)	0.012*	0.21 (0.05–0.67)	0.012*
CS 3	0.31 (0.04–1.94)	0.209	0.40 (0.05–2.49)	0.323
CS 4	1.79 (0.81–3.95)	0.148	1.07 (0.49–2.33)	0.857
CS 5	1.56 (0.71–3.55)	0.277	0.89 (0.43–1.90)	0.768
CS 6	1.21 (0.45–3.11)	0.697	1.14 (0.44–2.89)	0.781
CS 7	0.42 (0.18–0.99)	0.047*	0.28 (0.11–0.65)	0.004*
CS 8	0.38 (0.17–0.84)	0.017*	0.24 (0.11–0.54)	0.001*

* *p*-value less than 0.05

The accuracy of decisions was also evaluated according to combined educational levels and the completion of postgraduate training (Table 5). Participants who completed postgraduate training in cervical spine care in addition to their education made more appropriate treatment decisions. The correct decision rate was at least 50% for the CS 1 CM case, and the proportion of correct decisions was less than 30% for cases CS 2 and CS 3. Participants who had completed postgraduate training in the neck were significantly more likely to make appropriate decisions for cases CS 1 (95% CI 2.02–9.36, *p* = 0.001), CS 2 (95% CI 1.86-181.02, *p* = 0.030), CS 4 (95% CI 1.20–5.72, *p* = 0.016), and CS 7 (95% CI 1.21–8.89, *p* = 0.025) than participants who did not complete postgraduate training in the neck.

**Table 5:** Descriptive statistics. The ratio of acceptable and unacceptable answers based on the educational level and combination with the neck-specific postgraduate trainings

	BSc (*N* = 43)	MSc (*N* = 7)	BSc with PGTr (*N* = 58)	MSc with PGTr (*N* = 20)	Total (*N* = 128)
**CS 1**					
Acc	19 (44.2%)	2 (28.6%)	47 (81.0%)	12 (60.0%)	80 (62.5%)
UAcc	24 (55.8%)	5 (71.4%)	11 (19.0%)	8 (40.0%)	48 (37.5%)
**CS 2**					
Acc	1 (2.3%)	0 (0.0%)	10 (17.2%)	3 (15.0%)	14 (10.9%)
UAcc	42 (97.7%)	7 (100.0%)	48 (82.8%)	17 (85.0%)	114 (89.1%)
**CS 3**					
Acc	1 (2.3%)	0 (0.0%)	2 (3.4%)	2 (10.0%)	5 (3.9%)
UAcc	42 (97.7%)	7 (100.0%)	56 (96.6%)	18 (90.0%)	123 (96.1%)
**CS 4**					
Acc	24 (55.8%)	5 (71.4%)	44 (75.9%)	17 (85.0%)	90 (70.3%)
UAcc	19 (44.2%)	2 (28.6%)	14 (24.1%)	3 (15.0%)	38 (29.7%)
**CS 5**					
Acc	14 (32.6%)	3 (42.9%)	24 (41.4%)	4 (20.0%)	45 (35.2%)
UAcc	29 (67.4%)	4 (57.1%)	34 (58.6%)	16 (80.0%)	83 (64.8%)
**CS 6**					
Acc	34 (79.1%)	6 (85.7%)	49 (84.5%)	17 (85.0%)	106 (82.8%)
UAcc	9 (20.9%)	1 (14.3%)	9 (15.5%)	3 (15.0%)	22 (17.2%)
**CS 7**					
Acc	4 (9.3%)	2 (28.6%)	18 (31.0%)	5 (25.0%)	29 (22.7%)
UAcc	39 (90.7%)	5 (71.4%)	40 (69.0%)	15 (75.0%)	99 (77.3%)
**CS 8**					
Acc	11 (25.6%)	1 (14.3%)	20 (34.5%)	5 (25.0%)	37(28.9%)
UAcc	32 (74.4%)	6 (85.7%)	38 (65.5%)	15 (75.0%)	91 (71.1%)

For MSK cases, the detection of cervical radiculopathy (CS 4) was significantly associated with both McKenzie (95% CI 1.39–11.45, *p* = 0.014) and manual therapy (95% CI 1.53–20.86, *p* = 0.016) training. Decisions about CM cases (CS 1, CS 2, CS 7, and CS 8) were significantly influenced by continuing education in cervical spine stabilization training. The participants who attended McKenzie training were more likely to recognize the signs and symptoms of fracture (CS 1) (95% CI 1.22–7.28, *p* = 0.021) (Table 6).

**Table 6:** Impact of postgraduate training on the proportion of correct decisions in different cases

	McKenzie method		Cervical spine stabilization training		Manual therapy method	
	**OR (95% CI)**	***p*** **-value**	**OR (95% CI)**	***p*** **-value**	**OR (95% CI)**	***p*** **-value**
CS 1	2.84(1.22–7.28)	0.021*	3.26(1.22–10.36)	0.027*	1.44(0.61–3.64)	0.415
CS 2	2.01(0.62–6.24)	0.229	4.70(1.46–15.24)	0.009*	0.92 (0.20–3.22)	0.907
CS 3	0.60(0.03–4.26)	0.657	2.61 (0.33–16.59)	0.307	2.37 (0.30-15.01)	0.358
CS 4	3.64(1.39–11.45)	0.014*	0.81(0.33–2.07)	0.641	4.74(1.53–20.86)	0.016*
CS 5	1.18(0.53–2.59)	0.685	1.35 (0.56–3.22)	0.495	0.51 (0.19–1.26)	0.162
CS 6	1.47(0.53–4.78)	0.484	0.66 (0.24–2.02)	0.437	2.06 (0.64–9.25)	0.275
CS 7	1.72(0.70–4.08)	0.226	3.17 (1.25–7.99)	0.014*	0.86 (0.29–2.27)	0.774
CS 8	0.72(0.29–1.69)	0.467	2.43 (1.00-5.90)	0.049*	0.92 (0.35–2.25)	0.859

* *p*-value less than 0.05

## Discussion

This study evaluated the diagnostic decision-making skills of physiotherapists using eight case studies of cervical spine complaints. The findings demonstrated variability in clinical judgment; the majority of respondents selected appropriate treatment strategies for MSK cases, whereas a significantly smaller proportion of participants made correct decisions for cases categorized CM. These results suggest that participants have greater proficiency in identifying and managing MSK-related cervical conditions compared to CM cases, highlighting potential areas for targeted educational interventions. Studies have suggested that physical therapists are more likely to make appropriate treatment decisions for MSK problems within their field of professional activity. The participants in the study by Jette et al. (2006) made correct treatment decisions in 87.3% of MSK cases within their scope of practice. The physiotherapists assessed by Budtz et al. (2021) made correct treatment decisions in 42% of the MSK cases. The results of this study on accurate assessment and decision-making in MSK cases are consistent with previous research. Nearly two-thirds of participants made correct treatment decisions for both MSK cases; however, when evaluated individually, MSK cases, such as cervical radiculopathy and myofascial pain syndrome, resulted in even more favorable rates. Additionally, it is worth noting that previous studies have investigated the ability of physiotherapists to recognize and differentiate between musculoskeletal, critical musculoskeletal, and complex musculoskeletal cases. Nowadays, reflecting the worldwide shortage of physicians in healthcare, the question of whether physiotherapists can replace physicians in some regions of direct care and primary care in the musculoskeletal field, specifically in areas such as examination, triage, and management, is becoming increasingly prominent. In response to this question, numerous review publications indicate that advanced physiotherapists exhibit diagnostic accuracy and effective management of musculoskeletal patients that are closely comparable to those of orthopedic surgeons [46–48]. Similar to the results of the aforementioned results, this study’s findings showed that physiotherapists with higher qualifications (MSc or postgraduate training) were more likely to choose the correct treatment procedures in MSK cases.

Several studies have demonstrated that physiotherapists make less appropriate differential diagnoses for complaints requiring medical evaluation and immediate treatment [28, 30, 31]. In a study by Keller et al. (2022) in Switzerland, therapists recognized 62% of red flags. Ladeira (2018) reported that almost 52.7% of physiotherapists recognized the indication for a medical referral without physiotherapy intervention in cases with red-flag signs and symptoms. Contrary to Jette et al.‘s results (2006), in this study, less than a quarter of respondents made correct treatment decisions (with at least three correct answers) in the CM category. Regrettably, only one person correctly identified the contraindication to physical therapy in all cases. This result is similar to the results (5%) of Budtz et al. (2021).

However, the comparison of results between studies is significantly limited by the differences in cases; some studies included case reports of problems affecting other body regions (Ladeira, 2018 - lumbar spine) and cases from other specialties [28, 30, 31].

Ojha et al.‘s systematic review found statistically significant and clinically meaningful satisfaction and outcomes were better in cohorts receiving direct access physiotherapy than in those receiving referred episodes of care [3]. In the present study, we found no statistically significant difference in the appropriate treatment decisions for either MSK or CM cases, regardless of whether respondents treated patients through direct or indirect access to physiotherapy.

Our results revealed that the recognition rate of warning signs and symptoms of cervical spine complaints by physiotherapists was low. Notably, fewer than 10% of the participants in the study were treated for cervical spine complaints in their practice, which may partly explain the low rate of appropriate decisions. However, the development of physiotherapy as a science and advances in manual techniques have contributed to the development of physiotherapy methods unique to the cervical spine, whose anatomy, biomechanics, and physiology are different from those of the lumbar spine. The scientific background for different approaches and treatment procedures was first studied in the 1990s. Since the 2000s, clinical evidence has supported the effectiveness of specific assessment methods and therapeutic interventions [49–51].

Budtz et al. (2021) demonstrated a link between good decision-making and more than 5 years of experience, especially for the differential diagnosis of critical health conditions. In contrast, Jette et al. (2006) and Keller et al. (2022) reported no significant associations between correct decisions and professional experience (more than 10 years). This study did not detect a significant correlation between correct decision-making and the observed p-value for all cases; however, longer experience increases the odds of correct decision-making on a case-specific basis.

Two studies revealed a link between correct differential diagnosis, higher professional education, and orthopedic specialization [28, 30]. According to Jette et al. (2006), more therapists (62.2%) who specialized in orthopedics field correctly identified signs and symptoms of a critical health condition than therapists who are not specialized in orthopedics field (46.5%). Moreover, in Ladeira’s (2018) study, therapists who specialized in orthopedics recognized 53.5% of CM cases. In our study, the rate of recognition of the threatening signs and symptoms of cervical myelopathy (CS 2) was low among all the participants, but the participants with the most experience in orthopedics and traumatology were more likely to recognize red flags and make correct treatment decisions. Furthermore, significant results were obtained for the case related to cervical instability (CS 7) and the case related to the metastatic bone tumor (CS 8) in terms of choosing the right treatment. In this study, participants who specialized in trauma care recognized the signs and symptoms of fracture well. However, our results cannot be accurately compared with the results of Ladeira (2018) because the physiotherapists in his study had postgraduate specialist training in orthopedics. In Hungary, postgraduate specialization according to clinical fields is not possible.

A high proportion of participants made inappropriate treatment decisions for cervical myelopathy (CS 2) and angina pectoris (CS 3) in our study. In these two cases, the participating therapists did not consider referral to a doctor or medical examination/intervention necessary. The high rate of inappropriate decisions in these cases is concerning. Cervical myelopathy is a common degenerative condition with a high incidence of spinal compression symptoms, including difficulty walking and numbness and clumsiness in both hands. Life-threatening signs and symptoms indicate the need for immediate medical evaluation and intervention [52–56]. The other critical CM case was an angina pectoris case due to coronary artery disease (CS 3). Pain in the anterior neck is a less common presentation of angina pectoris [57]. The low rate of correct decisions for this case is concerning, but recognizing atypical symptoms while reading a case is difficult, and signs of myocardial infarction in the patient’s history may were insufficient. Our results revealed no significant differences based on the combination of educational level (BSc, MSc/PhD) and cervical spine postgraduate training for this case; thus, the effect was not due to differential diagnostic knowledge acquired through cervical spine postgraduate training. Postgraduate training provides physiotherapists with differential diagnostic knowledge, enabling them to make a comprehensive assessment of warning signs and symptoms.

Participants who mastered the McKenzie method could identify severe pathological conditions of the cervical spine in the fracture case (CS1), as the theoretical part of the training included red flags. The course “Examination and treatment of the cervical spine with special attention to segmental instability for physiotherapists” is an optional theoretical and practical accredited course in Hungary. This course covers the theoretical and practical knowledge of manual therapy according to G. D. Maitland [58]. Almost one-third of the therapists in this study received training in this area, and similar proportions of the therapists were trained in manual therapy to acquire differential diagnostic skills.

Our results support the hypothesis that the completion of postgraduate training in the treatment of the cervical spine positively impacts decision-making and the recognition of life-threatening signs and symptoms in CM cases. Professionals trained to manage musculoskeletal disorders of the neck have broader knowledge of physiotherapy, are more confident in making correct treatment decisions for musculoskeletal problems, and are more likely to recognize severe pathological cases. Our findings are consistent with the results of Shavit et al. (2025), who demonstrated the positive impact of training in medical screening and differential diagnosis on reducing physiotherapists’ concerns and increasing clinical confidence and self-efficacy [59].

### Limitations

This study has several limitations that should be acknowledged. First, the cross-sectional design limits the ability to draw predictive conclusions based on the results. Second, our questionnaire did not fully assess participants’ knowledge because they could not ask questions after reviewing the findings. The information gained in this manner is not equivalent to that achieved through face-to-face examinations. Additionally, the interpretability of our results is limited due to the critical health conditions in the case reports, which require knowledge of key warning signs and symptoms. Since our questionnaire focused on cases of cervical spine complaints, further studies are needed to get a more complete picture of the differential diagnostic decision-making ability of physiotherapists working in Hungary.

## Conclusions

To our knowledge, the effects of the differential diagnostic knowledge of Hungarian physical therapists in the recognition of warning signs and symptoms have not been previously studied. Although limited by self-report, cross-sectional design, the results of our study are particularly important because an increasing number of professionals trained in Hungary are working in private practices, where patients arrive without prior medical examination, and rapid consultation and referral of cases are not always possible.

In Hungary, the training outcome requirements for physiotherapists do not include differential diagnostic knowledge [60]. Moreover, to the best of our knowledge, no postgraduate training course in Hungary specifically teaches the process of differential diagnosis in physiotherapy, but differential diagnosis is part of the curriculum of some training courses. In line with the conclusions of international publications, our results suggest that an independent postgraduate course aimed at acquiring differential diagnostic thinking and skills should be added to the curriculum, contrary to the current practice in Hungary. However, we are pleased to share that, based on our results, BSc students will receive a 14-hour course on the basics of differential diagnosis starting in 2024 at the Faculty of Health Sciences and Social Studies of the University, with special emphasis on recognizing red flags and life-threatening conditions.

## Electronic supplementary material

Below is the link to the electronic supplementary material.

Supplementary Material 1

## Data Availability

The datasets used and/or analyzed during the current study are available from the corresponding author on reasonable request.

## Abbreviations

MSK: Musculoskeletal
CM: Critical Medical
WCPT: World Confederation of Physical Therapists
MESZK: Chamber of Hungarian Health Care Professionals
MGYFT: Association of Hungarian Physiotherapists
BSc: Bachelor’s Degree
MSc: Master’s Degree
CS: Case Study
ORs: Odds Ratios
95% CIs: Confidence Intervals
No.: Number
PGTr: postgraduate Training
Acc: Acceptable
UAcc: Unacceptable
id est=that is: i.e.
