# Supplementary material for: Assessment of differential diagnostic skills of physiotherapists related to the cervical spine - approaches to improving effectiveness: observational, cross-sectional study
Source: BMC Med Educ. 2025 Jul 16;25:1065. doi: 10.1186/s12909-025-07682-x (PMC12269254; doi:10.1186/s12909-025-07682-x)
Supplement: Supplementary file 1 — Supplementary Material 1 [file 12909_2025_7682_MOESM1_ESM.docx]

**Additional file**

**Appendix 1.** Data from respondents of the survey

This part of the survey includes 14 questions about your demographics, education and work experience. The questionnaire takes 2 minutes to complete. Please answer all questions if you can.

1. Age: __________

2. Sex:

- female
- male

3. When did you earn your bachelor's degree? (year) _________________________________

4. Where did you earn your first graduate degree? ___________________________________

5. What is your highest level of education as a physiotherapist?

- BSc
- MSc
- PhD
- Other: ________________________________________________________________

6. Have you participated in postgraduate training?

- yes
- no

If so, what kind of training was it? ____________________________________________

7. What courses have you attended to acquire knowledge in differential diagnosis?

________________________________________________________________________

8. Have you completed a course on cervical spine treatment?

- yes
- no

If so, which one? __________________________________________________________

9. How many years of practical experience do you have? _____________________________

10. Where have you worked in the past year? (You can tick more than one answer.)

- clinic/hospital: outpatient care
- clinic/hospital: inpatient care
- private practice
- other: ________________________________________________________________

11. What was the average number of patients you had per week in the past year?

- between 0 and 10
- between 11 and 20
- between 21 and 30
- between 31 and 40
- between 41 and 50
- between 51 and 60
- over 60

12. On average, what kind of problems do you deal with?

- orthopedics
- traumatology
- neurology
- neurosurgery
- pediatrics
- surgery
- other: _______________________________________________________________

13. Do patients usually come to you with a doctor's referral?

- yes
- no

14. What percentage of your caseload are patients presenting with neck pain?

- 0 %
- under 10 %
- 10 – 30 %
- 31 – 50 %
- over 50 %

**Appendix 2.** Cases with musculoskeletal problems and cases with warning signs and symptoms

| **MSK cases** |  |
| --- | --- |
| **CS 4** cervical radiculopathy (Wachidah et Herawati, 2021; Farhana et al, 2014) |  |
| **CS 6** myofascial pain syndrome (Jalil et al, 2010) |  |
| **CM cases** | **warning signs and symptoms** |
| **CS 1** odontoid fracture (Ross&Cheeks, 2008) | age, trauma, severe osteoporosis, tumor, limited mobility, severe pain on movement (VAS 9/10), pain persisting at rest, reduced sensation corresponding to C2-3 dermatome |
| **CS 2** cervical myelopathy (Smith et al, 2014) | age, complaint for 8 weeks, gait disturbance, clumsiness of both hands, brisk upper and lower limb reflexes |
| **CS 3** angina pectoris (Mathers, 2012) | pain on running/exercise, signs of myocardial infarction on prior ECG |
| **CS 5** vertebral artery dissection (Gomez-Rojas et al, 2020) | severe bilateral neck pain, dizziness after connective tissue massage, nausea/vomiting, numbness of both upper extremities |
| **CS 7** atlantoaxial instability (Bayer et al, 2021; Lyons et al, 2018) | neck pain for more than 1 year, progressively worsening, 13 years of RA, total limitation of cervical spine movement in all directions, pain, loss of control during traction |
| **CS 8** metastatic tumor (Chaniotis, 2012) | pain at night, weakness, difficulty getting up from a chair or going up the stairs, loss of sensation corresponding to C6 dermatome |

**Appendix 3.** Classification of management decisions

| **Provide advice, refer to another healthcare** | **Physiotherapeutic intervention** |
| --- | --- |
| - cervical collar  - bed rest | - massage, trigger point therapy  - electrotherapy |
| - cooling/heating | - neurodynamic mobilization |
| - imaging | - traction on the cervical spine |
| - referral to a physician without physiotherapy treatment | - cervical spine manipulation |
| - consultation with a psychologist | - mobilization exercises, preferred movement directions |
| - education about necessary lifestyle changes (posture and exercise to alleviate symptoms) | - muscle strengthening exercises, restoring the function of local stabilizers |
|  | - exercises to improve coordination |
|  | - endurance and strength exercises |
|  | - compiling a program of physiotherapy exercises to be done at home |
|  | - physiotherapy treatment and referral to other healthcare professional/physician |
